# ELSA and RRI – Editorial

**DOI:** 10.1186/s40504-014-0021-8

**Published:** 2015-01-29

**Authors:** Ellen-Marie Forsberg

**Affiliations:** Oslo and Akershus University College, Research Group on Responsible Innovation, P.O. Box 4 St. Olavs plass, 0130 Oslo, Norway

## Abstract

This editorial presents the background for the article collection ‘ELSA and RRI’. It sets the stage for the topics discussed in the collection and briefly presents the different contributions. It concludes by opening up for continued discussion of the relations between ELSA and RRI.

Many of the authors and readers of Life Sciences, Society and Policy (and the earlier Genomics, Society and Policy) have been involved in so-called ELSA studies, studies of Ethical, Legal and Social Aspects of scientific and technological developments. ELSA studies have been funded alongside major technology development programs, especially in biotechnology, since the Human Genome Project in 1990. The purpose has been to provide a knowledge base for developing emerging science and technologies in a responsible way and with an awareness of the ethical, legal and social aspects and impacts of such developments. ELSA studies have bordered on, and to an increasing extent included, Science and Technology Studies (STS), with a broader social and cultural perspective on the relation between science, technology and society.

ELSA studies have been criticised, both from researchers that identify with the ELSA agenda and from other researchers, for being simply an add-on to the large science and technology development projects (Balmer et al. 2012). It has been claimed that funding for ELSA, which has been relatively minor compared to funding for natural science and technology research, has simply been a PR move that keeps ELSA researchers busy and critical societal groups at bay, and do not affect science and technology development at all. As a response to this criticism there have been attempts at designing more integrated projects, where the ELSA researchers interact with the natural science and technology researchers in a way that is supposed to lead to mutual learning and affect the research on both sides (Fisher et al. 2012, Nydal et al. 2012). Good designs for such integrated research projects are still in the making and several projects explore ways to achieve good learning processes in the projects combined with production of relevant, publishable ELSA knowledge. Action research is a research tradition that appears to be in growth for achieving this dual end.

Alongside this development of ELSA research and integrated projects a new research and development agenda has gradually come to the fore. Responsible Research and Innovation (RRI) has been become prominent in European research and innovation policy, in particular with the new European Commission (EC) research funding programme Horizon 2020. Responsible Innovation (RI) initiatives and concepts are also developed in different forms all over Europe, and beyond, without necessarily referring to the European Commission RRI concept (see von Schomberg 2012 and Owen et al. 2012). Common for most RRI and RI approaches, though, is that they are practically oriented. In the official EC version practical ends such as gender balance and open access are included, and other approaches usually focus on reflective engagement of scientists and innovators with stakeholders or the public.

It is therefore of interest to discuss how ELSA research seems to fare related to this new practice oriented agenda. Will ELSA researchers convert to being facilitators for reflection among natural scientists? If so, are they really qualified for such group facilitation work? Will what has formerly been labelled as ELSA research in the future mainly be presented simply as contributions to the various disciplines, such as sociology, legal studies, philosophy or ethics, thus disintegrating the interdisciplinary concept of ELSA? Was ELSA a too artificially construed field in the first place? Or is there still a need for ELSA as an interdisciplinary field; for instance in providing a conceptual platform for RRI? Even if RRI is a development agenda, shaping science and technology development in a more societally robust direction, it does require more conceptual work, such as better accounting for the understanding of responsibility that is implied in the concept. In providing a conceptual platform for RRI ELSA research may further develop and fields that have not yet been prominent in ELSA studies, such as innovation studies, may become increasingly important. How can ELSA researchers adapt to the current developments in a way that both safeguards and further develops their key competencies?

Questions such as these were addressed in a Norwegian ELSA conference December 2012. The conference was co-organised by the Research group on Responsible Innovation at Oslo and Akershus University College and the Norwegian Research Council’s ELSA programme. The background for the workshop was that the ELSA programme is closing after two programme periods of altogether 12 years, and an important purpose of the conference was for the Norwegian ELSA researcher community to organise itself for upcoming challenges. The conference was organised as a dialogue conference in order to allow for more time for discussion than in most purely academic conferences. However, even with this specific national context it was clear that this situation is not unique to Norway. International key note speakers were therefore invited to present perspectives from the Netherlands and the UK, and the new European initiative on RRI was all the time present in the discussions. There seems to be a need for all ELSA researchers, no matter their national identities, to re-orient themselves in light of current developments, or at least to reflect anew on the basic assumptions in this field.

In this article collection some of the points that were addressed in the conference (either as key note presentations or as perspectives in the discussions) have been developed into papers. All the papers involve discussions about whether and how ELSA research can and should adapt to or contribute to RRI, from philosophical and sociological perspectives. Zwart et al. (2014) critically discuss how ELSA researchers in the paradigm of RRI become positioned more clearly as co-responsible for innovation. Myskja et al. (2014) argue that ELSA research for years have already had the diversity of practical and theoretical approaches currently requested and that there is no need to reject ELSA research in favour of a new approach. Forsberg (2014) discusses strategies the Norwegian ELSA researcher community may employ to survive without the ELSA programme. Oftedal et al. (2014) welcomes the RRI agenda and suggest that it opens up for recognising the importance of the philosophy of science more than classical ELSA research has seemed to do. Rip (2014) shows how RRI is a social innovation that should not only be understood in light of ELSA studies or other specific developments, but as representing a more general destabilisation of the divisions of moral labour across science and society.

Even if this article collection is a result of the Norwegian conference, the discussions about ELSA and RRI extend far beyond Norway and this conference. We therefore invite further contributions to this collection. ELSA research needs to be discussed and re-defined continuously and in particular vis-à-vis the notion of RRI, which now reconfigures the whole working space of European ELSA researchers.
